# Axon Guidance and Collective Cell Migration by Substrate-Derived Attractants

**DOI:** 10.3389/fnmol.2019.00148

**Published:** 2019-06-06

**Authors:** Hermann Aberle

**Affiliations:** Functional Cell Morphology Lab, Heinrich Heine University Duesseldorf, Duesseldorf, Germany

**Keywords:** axon guidance, collective cell migration, attraction, Sidestep, Netrin, SDF1

## Abstract

Neurons have evolved specialized growth structures to reach and innervate their target cells. These growth cones express specific receptor molecules that sense environmental cues and transform them into steering decisions. Historically, various concepts of axon guidance have been developed to better understand how axons reach and identify their targets. The essence of these efforts seems to be that growth cones require solid substrates and that major guidance decisions are initiated by extracellular cues. These sometimes highly conserved ligands and receptors have been extensively characterized and mediate four major guidance forces: chemoattraction, chemorepulsion, contact attraction and contact repulsion. However, during development, cells, too, do migrate in order to reach molecularly-defined niches at target locations. In fact, axonal growth could be regarded as a special case of cellular migration, where only a highly polarized portion of the cell is elongating. Here, I combine several examples from genetically tractable model organisms, such as *Drosophila* or zebrafish, in which cells and axons are guided by attractive cues. Regardless, if these cues are secreted into the extracellular space or exposed on cellular surfaces, migrating cells and axons seem to keep close contact with these attractants and seem to detect them right at their source. Migration towards and along such substrate-derived attractants seem to be particularly robust, as genetic deletion induces obvious searching behaviors and permanent guidance errors. In addition, forced expression of these factors in ectopic tissues is highly distractive too, regardless of the pattern of other endogenous cues. Thus, guidance and migration towards and along attractive tissues is a powerful steering mechanism that exploits affinity differences to the surroundings and, in some instances, determines growth trajectories from source to target region.

## Introduction—A Short Historical Perspective of Axon Guidance

Postmitotic neurons polarize and send out axons that make a series of sequential pathway choices to reach distant target cells. The molecular regulation of these outgrowth, steering and recognition processes became a fundamental topic in neuroscience during the last century. Ever since the discovery of the growth cone (“cono de crecimiento”) by Ramon y Cajal, neuroscientists wondered about the molecular nature of axon navigation and target recognition (Ramon Y Cajal, 1890; Raper and Mason, 2010).

In the cellular and histological era, anatomical studies and transplantation/extirpation experiments were the driving force for predominantly mechanical interpretations of nerve growth, based on the demonstration of a substrate requirement by Harrison or the concept of “contact action” along topographic features by Weiss (Harrison, 1907, 1910; Weiss, 1941). Sperry, on the other hand, developed an independent hypothesis, proclaiming that neuronal networks organize on the basis of specific “cytochemical affinities” (Sperry, 1963). Subsequent investigations of developing sensory nerves in grasshopper limb buds formed the idea that sheathing cells (glia) at stereotyped positions constitute a “system of signposts” with directive activities (Bate, 1976). Similar spatial arrangements were detected by Singer and co-workers in the late 1970s. Their histological studies in developing newts revealed gaps between neural progenitors of the ventricular zone that became filled with axon bundles over time, giving rise to the “blueprint hypothesis,” i.e., axons migrate along preformed “channels” that serve as mechanical guides (Singer et al., 1979; Gottlieb, 1980). Pre-existing substrate routes were also deduced from transplanting eyes to non-optic regions, including tails, of tadpoles, as ectopic axons followed specific pathways to the brain (Katz and Lasek, 1978, 1979). However, mechanical factors and physical features alone cannot be entirely responsible for wiring highly complex nervous systems with billions of nerve connections.

In fact, the upcoming genetic and molecular era helped to uncover biochemical cues controlling axon guidance. Staining grasshopper embryos with monoclonal antibodies showed that axon tracks in the central nervous system (CNS) express specific surface molecules that label subsets of axonal pathways for selective recognition by outgrowing growth cones (Goodman et al., 1982). These identification tags were proposed to be recognized selectively by adequately specified growth cones, giving rise to the “labeled pathways hypothesis” that was strongly supported by steering decisions of identifiable axons and direct experimental manipulations such as single cell ablations (Goodman et al., 1984).

The surge of recombinant DNA technology and the execution of genetic screens in the 1980s and 1990s led to the identification of conserved guidance molecules that function as secreted ligands or transmembrane receptors and attract or repel axons (Tessier-Lavigne and Goodman, 1996; Dickson, 2002). Thus, the bottom line from this historical perspective is that growing nerves require a substrate that is selected based on the steering information of an exquisite set of extracellular proteins. In the following, I will continue predominantly with substrate-derived attractants, molecules secreted from or presented on cells that simultaneously serve as a preferred growth substrate. For more general aspects of axon guidance, in particular, repulsion, see excellent reviews by Kolodkin and Tessier-Lavigne (2011) and Seiradake et al. (2016).

## Substrate Recognition and Axon Sorting by Adhesive Interactions

Growth cones indeed carefully choose their substrates and are very well able to discriminate between different surfaces both in tissue culture plates (Letourneau, 1975b) and in selective choice assays testing target from non-target cells (Bonhoeffer and Huf, 1980). In patterned plastic dishes with different molecules in grid-like arrangements, chick sensory neurons preferentially elongated on substrates with increased adhesiveness (Letourneau, 1975a). Growth cones explored but did not cross onto non-adhesive areas (Letourneau, 1975a; Oakley and Tosney, 1993). In fact, differential adhesion guided axons into narrow channels with increased adhesiveness (Hammarback et al., 1985). However, other studies found no or little correlations between the relative substrate adhesiveness and growth rates or the molecular composition of substrates and preferential growth cone selection, respectively (Lemmon et al., 1992).

Nevertheless, direct contact to a substrate is clearly necessary for sustained axon growth, and the formation of local adhesion plaques might influence guidance decisions. Single filopodia in pioneering axons have been shown to trigger growth cone turning of chick sensory neurons towards laminin-coated beads (Kuhn et al., 1998). Here, growth cones grew out on a uniform fibronectin substrate and encountered a laminin-coated bead held in place by optical trapping using a laser tweezer. Filopodia explored the bead before forming an initial stable contact, which then attracted the entire growth cone (Kuhn et al., 1998). Similar observations have been made in Grasshopper embryos *in vivo* where turning of pioneering growth cones in the Ti1 sensory pathway towards guidepost cells was initiated by filopodial contacts (O’Connor et al., 1990). Importantly, differential expression of a single Cadherin adhesion protein was necessary and sufficient for sorting axonal processes in the fly visual system (Schwabe et al., 2014). Similar to groups of cells that sort themselves out based on differential adhesion, for example during the formation of embryonic compartment boundaries (Amack and Manning, 2012), establishing adhesive contacts to a preferred substrate could be an important guidance principle.

## Axons and Migrating Cells Are Subject to Similar Steering Principles

**Figure 1:**
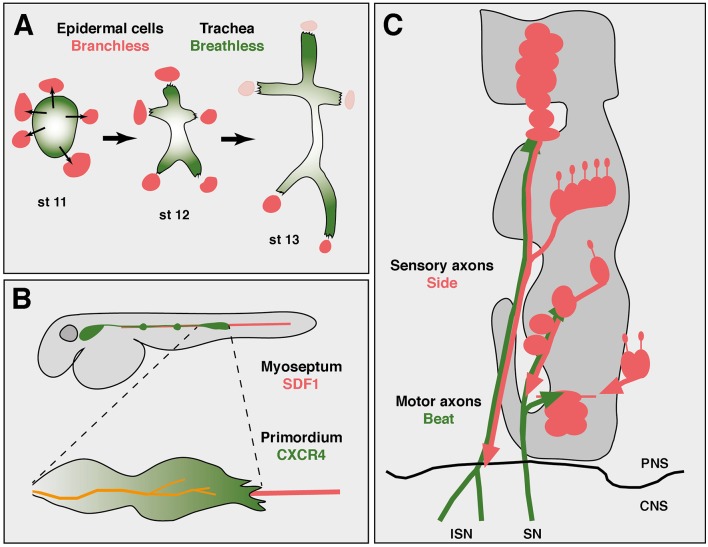
Axon guidance and cell migration towards and along attractive substrates. **(A)** Collective cell migration during development of the tracheal system in an abdominal hemisegment of *Drosophila* embryos. Simplified scheme showing the development of tracheal branches from a cluster of coherent precursors (green) in the dorsal epidermis. At developmental stage 11 (st 11), five cell clusters outside the tracheal anlage express Branchless (Bnl, red), which induces tracheal outgrowth (thin arrows). At stage 12, Breathless-expressing tracheal cells recognize and grow towards Bnl-expressing cells. Retreat of the Bnl source extends these initial branches. At stage 13, the dorsal-most branches reach their final positions at the dorsal midline and segmental borders, where Bnl expression eventually ceases (pink). Individual cells are not resolved in this scheme. Thick arrows indicate developmental progression. Anterior is left, dorsal is up. **(B)** Axon “towing” by a migrating cell cluster during zebrafish development. The lateral line primordium (green) migrates from a placode near the head to its final destination in the tail, strictly following the horizontal myoseptum (red). Two neuromasts have already been deposited along its path (green dots). Leading cells in the primordium sprout long filopodia and express CXCR4 receptors that recognize the attractant SDF1 emanating from the substrate. The lateral line nerve (orange) co-migrates with the primordium and connects neuromasts to the brain. **(C)** Axon guidance along attractive substrates. Scheme of motor axon development in *Drosophila embryos* at stage 14. Motor axons of the intersegmental (ISN) and segmental (SN) nerve express Beaten path (green) and fasciculate with Sidestep-expressing sensory axons (red). Developing muscle fields do not express Sidestep at this stage and are outlined in gray. The central nervous system (CNS)/PNS boundary is marked (black line). Not to scale. Schemes according to Sutherland et al. (1996), Gilmour et al. (2004) and Siebert et al. (2009).

It is interesting to note that axon growth could be regarded as a specialized form of cell migration. By leaving the soma in place and extending only a portion of the cell, axonal growth might utilize principles established at the leading edge of migrating cells (von Philipsborn and Bastmeyer, 2007). Similar to growing axons, migrating cells, either as individuals or cohesive groups, depend on substrates and extracellular steering cues. During *Drosophila* development, for example, border cells, peripheral glial cells or tracheal cells, all show migration patterns along stereotypic pathways (Pocha and Montell, 2014). The tracheal system develops from clusters of coherent cells that invaginate from the ectoderm into the interior of the embryo (Figure 1A). These cells are all tightly connected with each other by adherence junctions. Specialized cells at the leading tip sprout motile filopodia, which recognize and follow local sources of the attractive factor Branchless (Bnl), a member of the fibroblast growth factor (FGF) family (Affolter and Caussinus, 2008). Bnl is expressed in non-tracheal cells consistently located ahead of elongating branches and is recognized by the Breathless (Btl) receptor expressed in trachea (Sutherland et al., 1996; Caussinus et al., 2008). Simultaneous labeling of Bnl source cells and Btl receiving cells revealed a dynamically moving signal source that directed the growth of tracheal branches (Du et al., 2017). Tip cells hence follow Bnl-expressing cells and thereby elongate the branch. Imaging Bnl-GFP fusion proteins during imaginal disc development demonstrated that Btl-expressing tracheal cells directly contact Bnl-GFP-expressing source cells (Sohr et al., 2019). Due to adhesive intercellular junctions that connect tip cells and stalk cells, the entire elongating branch resembles somehow, metaphorically speaking, an “axon-like” structure.

In fact, there are similar tip cell-guided collective migration events in vertebrates, too. Migration of cohesive cells has been observed during sprouting of blood vessels, invasion of cancer cells or development of neural crest cells, to name a few examples (Friedl and Gilmour, 2009; Szabó and Mayor, 2018). The attractants are not always known, but the chemokine SDF1 (stromal cell-derived factor 1, also called CXCL12), has been shown to play an important role as a substrate-derived attractant for migratory cells during vertebrate development, particularly in zebrafish (Doitsidou et al., 2002; Chalasani et al., 2003; Knaut et al., 2003). SDF1 is expressed along the horizontal myoseptum, amongst other regions, and directs the migration of the posterior lateral line primordium and its accompanying nerve (Figure 1B; Haas and Gilmour, 2006). The primordium consists of a placode-derived cell cluster and deposits small cell aggregates in regular intervals that develop into neuromasts, mechanosensory organs detecting water flow in fishes (Ghysen and Dambly-Chaudière, 2004).

Interestingly, the lateral line nerve, which innervates these neuromasts, maintains a central position in the advancing primordium and strikingly appears to co-migrate with it, a phenomenon that has been called “nerve towing” (Weiss, 1941; Gilmour et al., 2004). The primordium expresses the chemokine receptor CXCR4 that detects SDF1 (Peled et al., 1999; David et al., 2002). It is most strongly activated in tip cells at the leading edge and required for migration along SDF1 secreting substrates (Haas and Gilmour, 2006). Interruptions in the linear expression pattern or forced expression of SDF1 in ectopic tissues caused extremely abnormal migration patterns followed by erroneous distribution of neuromasts (Haas and Gilmour, 2006). Similarly, mutations in CXCR4 largely abolished the migration of the primordium and thus outgrowth of the lateral line nerve (Gilmour et al., 2004), indicating that SDF-1 ligands emanating from the horizontal myoseptum delineate a robust trajectory that guides the primordium, which in turn “pulls” the lateral line nerve from its origin in a cranial ganglion to the tail. While the molecular connections of the growth cone to cells in the primordium are unknown, this unusual guidance principle demonstrates a strict dependence on a substrate-derived attractant.

Although SDF1 is a small and secreted protein, and thus difficult to detect in tissues, it might not necessarily act over long distances but might locally enrich in the extracellular matrix as a short-range cue. First, it is stunning that, *in vivo* and in different organisms, attracted cells usually follow the dynamic expression domains of SDF1 very tightly (Doitsidou et al., 2002; Knaut et al., 2003). In this respect, it is important to note that in a developing embryo several tissues secrete SDF1 at the same time. For example, during zebrafish somitogenesis, SDF1 is expressed in somites, specific regions in the head and along the border of the trunk mesoderm (Doitsidou et al., 2002). Unrestricted diffusion from all these sources at the same time should distribute SDF1 widely in the body cavity, possibly interfering with gradient formation. But SDF1 signals from different sources do not seem to interfere with each other during normal development. Indeed, neither migrating cells nor axons are confused or distracted from neighboring SDF1 sources, indicating that its acts rather locally (Lewellis and Knaut, 2012; Lewellis et al., 2013). In addition, immunohistochemical stainings using specific polyclonal antibodies show cellular but not extracellular patterns in the mouse brain (Miller et al., 2005). Furthermore, the distribution of fluorescent SDF1-fusion proteins expressed from genomic BAC clones closely resembled that observed by *in situ* hybridizations (Bhattacharyya et al., 2008). It is possible that widespread diffusion is prevented by receptor-mediated clearance (Boldajipour et al., 2008) or proteoglycan trapping (Reiss et al., 2002). Taken together, available evidence supports the possibility of local SDF1 accumulations in the extracellular matrix of expressing cells.

## Axon Guidance Along Marked Substrates

Similar cell surface enrichments might also apply for Netrin-expressing cells. Secreted Netrin proteins are generally believed to attract commissural axons over long distances towards and across the midline. In the developing spinal cord, Netrin1 is expressed at highest levels in the floor plate, and hypomorphic gene-trap mice first revealed that it is required there to cross the midline (Serafini et al., 1996). Removing Netrin1 completely, in null mutant mice, resulted in an even stronger phenotype, with commissural axons rarely crossing the midline at all (Bin et al., 2015; Yung et al., 2015). Interestingly, conditional knockouts lacking Netrin1 selectively in floor plate cells of the hindbrain showed surprisingly little crossing defects. In contrast, deletions in neuronal progenitors of the ventricular zone prevented midline crossing, and the phenotypes were indistinguishable from null mutants. Netrin1 was detected on neuronal processes extending to the pial surface suggesting that commissural axons detect the protein there locally (Dominici et al., 2017; Varadarajan et al., 2017). Latest experiments in the spinal cord similarly found evidence for a haptotactic function of Netrins but chemoattraction from the floor plate also played a role (Moreno-Bravo et al., 2019; Wu et al., 2019). In fact, evidence for short-range function of Netrins was also obtained *in vitro* and in invertebrates. Netrin-coated beads triggered traction forces and reoriented spinal commissural axons by adhesive interactions* in vitro* (Moore et al., 2009). In addition, experiments in *Drosophila* showed that a membrane-tethered form of endogenous NetrinB rescued commissure formation indicating that Netrin secretion was not required (Brankatschk and Dickson, 2006). Future experiments should, therefore, address the exiting question if Netrins function as substrate-derived attractants.

Direct contact-dependent adhesion to an attractive cue was recently demonstrated in* C. elegans* and underlies the construction of a sensory circuit responding to harsh mechanical stimuli (Chen et al., 2019). Primary dendrites of the enormous PVD neuron adhere to and migrate along axons of the ALA interneuron *via* adhesive Sax7/L1CAM-Sax3/Robo interactions. ALA axons run along the lateral nerve cord and express the adhesion protein Sax7/L1CAM. Primary PVD dendrites, on the other hand, express Sax3/Robo and project along ALA axons (Ramirez-Suarez et al., 2019). Sax7/L1CAM co-immunoprecipitated with tagged Sax3/Robo proteins, and mutual recognition *in vivo* caused morphological changes in PVD dendritic growth cones and re-oriented their actin cytoskeleton (Chen et al., 2019). Substrate adhesion therefore correlated well with structural changes in the underlying cytoskeleton. Such substrate-mediated cytoskeleton remodeling has been observed in various neuronal motility systems (Suter and Forscher, 2000; Myers et al., 2011). Reorganization is usually mediated by transmembrane receptors and specialized cytoplasmic adaptor proteins thought to function as molecular clutches (Lin and Forscher, 1995; Bard et al., 2008; Myers et al., 2011). Dynamic linkage of guidance receptors to filopodial actin flow is, therefore, a possible scenario for the conversion of attractive cues into forward movements or growth cone turning.

Substrate-derived attractants also play an important role during *Drosophila* development. There is accumulating evidence that Sidestep (Side) marks permissive substrates for outgrowing motor axons (Figure 1C). Side is a transmembrane protein of the immunoglobulin family and attracts motor axons (Sink et al., 2001; de Jong et al., 2005). Motor axons pioneering the intersegmental nerve (ISN) leave the ventral nerve cord by growing along an array of Side-expressing cells (Siebert et al., 2009). Once in the periphery, they fasciculate with Side-positive, peripheral sensory axons that grow into the CNS. Although Side is difficult to detect in a subset of sensory axons, based on the location of the sensory clusters in the lateral body wall, major motor nerves could in principle reach their appropriate target regions by simply growing along sensory tracks followed by defasciculation into the muscle fields. Motor axons are firmly attached to sensory axons in wild-type embryos but show detachments in *side* mutants (Siebert et al., 2009). Beaten path Ia (Beat), also a member of the immunoglobulin family, is expressed in motor neurons and functions to detect Side (Fambrough and Goodman, 1996; Siebert et al., 2009). First, loss of *beat* leads to highly similar axon guidance errors and muscle innervation phenotypes as observed in *side* mutants (Fambrough and Goodman, 1996; Sink et al., 2001). A phenotype that is not increased in double mutants (Siebert et al., 2009). Second, Beat and Side interact with each other in S2 cell aggregations assays and in immunoprecipitation experiments (Siebert et al., 2009). Third, while Side is no longer detectable on peripheral nerves at the end of embryogenesis using Side-specific antibodies, it is constitutively expressed in *beat* mutants (Siebert et al., 2009). In fact, homozygous *beat* mutant embryos can be visually distinguished from heterozygous embryos based on Side expression, indicating that there is some sort of cross-regulation. And fourth, Side loses its ability to attract motor axons in *beat* mutants (Siebert et al., 2009). These results indicate that Beat on motor axons recognizes Side in substrates during the establishment of neuromuscular circuits.

*Drosophila* peripheral nerves express several axon guidance receptors of the immunoglobulin superfamily, but in direct comparisons, Side seems to be most potent in attracting motor axons (Kinold et al., 2018). In fact, overexpression of Side, but not the homophilic adhesion proteins Fasciclin II or Neuroglian, in developing muscles irreversibly attracted dorsally-directed ISN motor axons to ventral and lateral muscle precursors (Kinold et al., 2018). The *Drosophila* genome contains several paralogs of Beat and Side, and recent interactome assays revealed that several family members form ligand-receptor pairs or are expressed in synaptic partner neurons (Özkan et al., 2013; Tan et al., 2015). In addition, the expression patterns of some proteins in the Side family are consistent with a possible role as substrate-based cues (Li et al., 2017). Thus, proteins of the Beat and Side family might mediate contact attraction of various pathways, highlighting the importance of the spatiotemporal expression pattern and the subcellular localization of the proteins during axon guidance by attraction.

## Conclusion

Labeling cell surfaces along migratory routes with potent attractants might be a powerful means to steer cells and axons, irrespective of the nature of other factors nearby. Differential attraction, that is, preferred attraction to designated substrates over their surroundings, might actually be sufficient to guide cells and axons. However, I emphasize that due to space constraints other aspects of axon guidance and cell migration, such as repellents (Kolodkin and Tessier-Lavigne, 2011; Seiradake et al., 2016) or the relevance of birth order-dependent axon organization (Kulkarni et al., 2016), axon branching (Kalil and Dent, 2014) and new developmental rules for axon sorting (Langen et al., 2015) could not be covered. Largely unresolved remain also the signaling mechanisms downstream of adhesion receptors and the integration of attractive and repulsive cues in the growth cone. A highly interesting point is the consequence of axon guidance errors for adult locomotion and behavior. While several axon guidance diseases have been identified in humans (Engle, 2010), there are still only a few examples of inherited mutations affecting the wiring of the musculoskeletal system.

## Author Contributions

Concept, figure and writing by HA.

## Conflict of Interest Statement

The author declares that the research was conducted in the absence of any commercial or financial relationships that could be construed as a potential conflict of interest.
